# Supplementary material for: Perspectives of providing magnesium sulfate to patients with preeclampsia and eclampsia: A qualitative study amongst nurse-midwives in Dar es Salaam, Tanzania
Source: PLoS One. 2024 Aug 7;19(8):e0308382. doi: 10.1371/journal.pone.0308382 (PMC11305528; doi:10.1371/journal.pone.0308382)
Supplement: S1 Table — The authors extend their sincere gratitude to the Muhimbili University of Health and Allied Sciences for providing logistic support. Our thanks go to hospital administration for their support during data collection and study participants for participating in the study. We would also like to acknowledge Kornel Izdory Metheo for assisting with transcription and recruitment of participants. (DOCX) [file pone.0308382.s001.docx]

**Perspectives of providing magnesium sulfate to patients with preeclampsia and eclampsia: A qualitative study amongst Nurse-midwives in Dar es Salaam, Tanzania**

| **CODES** | **SUB CATEGORY** | **CATEGORY** |
| --- | --- | --- |
| - Severe features of preeclampsia | Indications for magnesium sulfate use | **Knowledge of magnesium sulfate provision** |
| - Protein in urine |  |  |
| - High blood pressure |  |  |
| - Convulsions |  |  |
|  | |  |
| - Dosing of magnesium sulfate | Determination of dosage regimen |  |
| - Dilution of magnesium sulfate |  |  |
| - Route of administration |  |  |
| - Dosing frequency |  |  |
| - Timing of administration |  |  |
| - Recurrent fits |  |  |
|  | |  |
| - Urine output and color | Monitoring of patient response |  |
| - Fetal heart rate |  |  |
| - Vital sign check |  |  |
| - Signs of toxicity |  |  |
| Effects of magnesium sulfate on monitoring |  |  |
| - Causes intoxication |  |  |
| - Suffocates the baby |  |  |
| - Nausea and vomiting |  |  |
| - Drowsiness |  |  |
| - Bleeding complications |  |  |
| - Pain and burning |  |  |
| - Prolongation of labor |  |  |
| - Operative deliveries |  |  |
|  | |  |
| - Impending childbirth | Postponement of magnesium sulfate administration |  |
| - Strong uterine contractions |  |  |
| - Respiratory depression |  |  |
| - Loss of tendon reflexes |  |  |
| - Low urine output |  |  |
| - Low blood pressure |  |  |
|  | | |
| - Saves babies' life | Benefits of using magnesium sulfate | **Reasons for magnesium sulfate use** |
| - Saves woman’s life |  |  |
| - Prevent convulsions |  |  |
| - Reduces pressure |  |  |
| - Safe to use |  |  |
| - Fast relief |  |  |
|  | |  |
| - Working experience | Confidence with own skill to use magnesium sulfate |  |
| - Knowledgeable |  |  |
| - Adequate preparation |  |  |
| - Individual commitment |  |  |
|  | | |
| - Lack of confidence | Individual barriers | **Barriers to magnesium sulfate provision** |
| - Inadequate knowledge |  |  |
| - Infrequent patient encounter |  |  |
| - Fear of side effects |  |  |
| - Frightening eclamptic patients |  |  |
|  | |  |
| - Inadequate training | Institutional barriers |  |
| - Underutilization of guidelines |  |  |
| - Shortage of staff |  |  |
| - Unsupportive infrastructures |  |  |
| - Shortage of equipment |  |  |
|  | | |
