# Supplementary material for: Perspectives of providing magnesium sulfate to patients with preeclampsia and eclampsia: A qualitative study amongst nurse-midwives in Dar es Salaam, Tanzania
Source: PLoS One. 2024 Aug 7;19(8):e0308382. doi: 10.1371/journal.pone.0308382 (PMC11305528; doi:10.1371/journal.pone.0308382)
Supplement: Attachment — Submitted filename: PE.docx [file pone.0308382.s002.docx]

**Dear Editor,**

I appreciate the chance to review the article titled: "Perspectives of providing magnesium sulfate to patients with preeclampsia or eclampsia: A qualitative study amongst Nurse-midwives in Dar es Salaam, Tanzania." The authors delve into a critical subject concerning health professionals' perceptions of magnesium sulfate administration to patients, which holds significant implications for positive pregnancy outcomes. Below are my recommendations for enhancing the quality of this study.

**Abstract**

the authors used an abbreviation of PE/E in the abstract you have to define it before you use it.

**Result section**

The authors are establishing thematic categories, like "Indications for magnesium sulfate use," solely relying on the perspective or opinion of a single individual. How did you ensure the validity of each thematic area, such as "Indications for magnesium sulfate use," when it's based solely on the perception or opinion of one individual? I recommend incorporating perspectives from other individuals regarding each thematic area.

Conclusion

Did the authors conclude based on participant reports or established facts that "Overall, magnesium sulfate is safe and beneficial for women and their babies when administered appropriately by nurse-midwives"? It would be preferable to conclude based on findings that align with the research objectives.
