# Supplementary material for: Perspectives of providing magnesium sulfate to patients with preeclampsia and eclampsia: A qualitative study amongst nurse-midwives in Dar es Salaam, Tanzania
Source: PLoS One. 2024 Aug 7;19(8):e0308382. doi: 10.1371/journal.pone.0308382 (PMC11305528; doi:10.1371/journal.pone.0308382)
Supplement: Attachment — Submitted filename: Response to Reviewers.docx [file pone.0308382.s003.docx]

**Perspectives of providing magnesium sulfate to patients with preeclampsia and eclampsia: A qualitative study amongst Nurse-midwives in Dar es Salaam, Tanzania**

**Response to reviewers’ comments**

We would like to thank all the reviewers for their valuable comments, which have significantly improved our work. We have revised the entire manuscript and addressed each comment point by point, as detailed in the table below.

| **Sn** | **Reviewer’s Comments** | **Authors’ Response** |
| --- | --- | --- |
| **Editor’s Comments** | | |
| 1 | Kindly rework the entire manuscript, especially the result section to have more than one perspective (quotes) for every theme/subtheme; the discussion & conclusion sections need to be more rigorous and extensive, containing policy or strategic implications of your findings | Thank you for your comment. We have reworked the entire manuscript, revised the result, discussion and conclusion sections. |
| 2 | Please include your full ethics statement in the ‘Methods’ section of your manuscript file. In your statement, please include the full name of the IRB or ethics committee who approved or waived your study, as well as whether or not you obtained informed written or verbal consent. If consent was waived for your study, please include this information in your statement as well. | Thank you for your comment. We have added a full ethics statement in the ‘Methods’ section of our manuscript file. We obtained written consent from participants and this information has been included as well. |
| **Reviewers’ Comments** | | |
| 1 | Is the manuscript technically sound, and do the data support the conclusions?  Reviewer #2: Partly | We appreciate your comment, we have revised the conclusion accordingly |
| 2 | Has the statistical analysis been performed appropriately and rigorously?  Reviewer #1: No | Thank you for your comment. However, we could not perform statistical analysis because this was a qualitative study. |
| 3 | 3. Have the authors made all data underlying the findings in their manuscript fully available?  Reviewer #2: No | We accept your comment, we have uploaded our codebook as supplementary information. |
| 4 | Is the manuscript presented in an intelligible fashion and written in standard English?  All reviewers: yes | Thank you for the comment |
|  |  |  |
| **Reviewer No 1** | | |
| Comment 1 | **Abstract:** mention: preeclampsia or eclampsia should: "and" as they are different entities. | Thank you for your comment, pre-eclampsia or eclampsia has been changed to pre-eclampsia and eclampsia. |
| Comment 2 | **Design of the study:** should mention "Questionnaire." method of research. | We appreciate your comment. However, this study employed an exploratory qualitative design and utilized in-depth interviews to gather qualitative data. An interview guide (questionnaire) with open-ended questions was used, as detailed in the Data Collection Methods and Guides section. |
| Comment 3 | **Statistical analysis:** much is needed to explain regarding the statistical methods used and the results extracted from the analysis. | Thank you for your comment. As this was a qualitative study, no statistical tests could be performed. Instead, the textual data were analyzed using qualitative content analysis. However, we have conducted descriptive statistical analysis specifically on the demographic characteristics of the participants. |
| Comment 4 | **References:** As the study is quite old one and has been investigated extensively before, **I wish the authors to gather and cite a more recent references** (2021/2022/2023 and 2024). | Thank you for your comment. We have updated our references to include more recent sources. However, we retained a few references from 2017, 2018, and 2019 because we could not find most relevant studies to replace them |
| **Reviewer #2:** | | |
| Comment 1 | The manuscript presents original research. However, the explanation of the **methodological design** seems weak and the **data collection process** needs to be better explained. | Thank you for your comment we have updated the justification to the chosen design to improve the explanation on methodological design.  The data collection process has been split to data collection tools and data collection procedures to improve clarity. More explanations have been added as well. |
| Comment 2 | The manuscript still lacks a clear statement **on data availability.** | We appreciate your comment. We have reviewed our statement; data has been made available as supplementary information. |
| Comment 3 | In terms of clarity, there are some **grammatical inconsistencies, but nothing serious.** | Thank you for your comment |
| **Reviewer #3:** | | |
| Comment 1 | **Abstract**  The authors used an abbreviation of PE/E in the abstract you have to define it before you use it. | The abbreviation PE/E has been omitted from the abstract. |
| Comment 2 | **Result section**  The authors are establishing thematic categories, like "Indications for magnesium sulfate use," solely relying on the perspective or opinion of a single individual. How did you ensure the validity of each thematic area, such as "Indications for magnesium sulfate use," when it's based solely on the perception or opinion of one individual? I recommend incorporating perspectives from other individuals regarding each thematic area. | Thank you for your feedback. We have revised the results section to incorporate viewpoints from various individuals. |
| Comment 3 | **Conclusion**  Did the authors conclude based on participant reports or established facts that "Overall, magnesium sulfate is safe and beneficial for women and their babies when administered appropriately by nurse-midwives"? It would be preferable to conclude based on findings that align with the research objectives. | We appreciate the comment. We have reworked the manuscript and made conclusion based on our findings. |
|  |  |  |
